# Pharmacological inhibition of epidermal growth factor receptor attenuates intracranial aneurysm formation by modulating the phenotype of vascular smooth muscle cells

**DOI:** 10.1111/cns.13735

**Published:** 2021-11-02

**Authors:** Yin Luo, Haishuang Tang, Zhaolong Zhang, Rui Zhao, Chuanchuan Wang, Wenguang Hou, Qinghai Huang, Jianmin Liu

**Affiliations:** ^1^ Department of Biomedical Engineering School of Life Science and Technology Huazhong University of Science and Technology Wuhan China; ^2^ Department of Neurosurgery Changhai Hospital Second Military Medical University Shanghai China; ^3^ Department of Neurology Strategic Support Force Medical Center of PLA Beijing China

**Keywords:** epidermal growth factor receptor, intracranial aneurysm, phenotypic modulation, vascular remodeling, vascular smooth muscle cell

## Abstract

**Aim:**

To study the effect of pharmacological inhibition of epidermal growth factor receptor (EGFR) on intracranial aneurysm (IA) initiation.

**Methods:**

Human IA samples were analyzed for the expression of p‐EGFR and alpha smooth muscle actin (α‐SMA) by immunofluorescence (IF). Rat models of IA were established to evaluate the ability of the EGFR inhibitor, erlotinib, to attenuate the incidence of IA. We analyzed anterior cerebral artery tissues by pathological and proteomic detection for the expression of p‐EGFR and relevant proteins, and vessel casting was used to evaluate the incidence of aneurysms in each group. Rat vascular smooth muscle cells (VSMCs) and endothelial cells were extracted and used to establish an in vitro co‐culture model in a flow chamber with or without erlotinib treatment. We determined p‐EGFR and relevant protein expression in VSMCs by immunoblotting analysis.

**Results:**

Epidermal growth factor receptor activation was found in human IA vessel walls and rat anterior cerebral artery walls. Treatment with erlotinib markedly attenuated the incidence of IA by inhibiting vascular remodeling and pro‐inflammatory transformation of VSMC in rat IA vessel walls. Activation of EGFR in rat VSMCs and phenotypic modulation of rat VSMCs were correlated with the strength of shear stress in vitro, and treatment with erlotinib reduced phenotypic modulation of rat VSMCs. In vitro experiments also revealed that EGFR activation could be induced by TNF‐α in human brain VSMCs.

**Conclusions:**

These results suggest that EGFR plays a critical role in the initiation of IA and that the EGFR inhibitor erlotinib protects rats from IA initiation by regulating phenotypic modulation of VSMCs.

## Graphical abstract

Epidermal growth factor receptor (EGFR) activation was found in human intracranial aneurysm tissues and rat intracranial aneurysm models. Pharmacological inhibition of EGFR by erlotinib attenuates intracranial aneurysm through regulating phenotype of vascular smooth muscle cells.

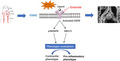

## 1 INTRODUCTION

Intracranial aneurysm (IA) is the leading cause of subarachnoid hemorrhages and portends severe morbidity and high mortality.^1^, ^2^ A cross‐sectional study conducted in Shanghai, China, found that the morbidity rate for IA was as high as 8.8%.^3^, ^4^ Surgical interventions include endovascular coiling and neurosurgical clipping and are the primary treatments for unruptured IAs; however, surgery leads to additional complications and the recurrence rate is relatively high.^5^, ^6^ Although considerable effort has been undertaken to develop methods to treat IA,^7^, ^8^, ^9^ there remains a lack of safe, effective, and noninvasive therapies.^10^, ^11^, ^12^ Therefore, it is important to seek appropriate noninvasive treatment strategies for IAs, including development of drug targets as novel new therapies against IA.

The occurrence of IA results from pathological processes, such as inflammatory responses and abnormal vascular remodeling.^11^, ^13^ Previous studies have revealed that abnormal vascular remodeling, the most important factor leading to the pathogenesis of IA, is caused by luminal forces that include shear stress, high blood flow, and turbulence.^14^, ^15^ In the early stages of aneurysm formation, the elevation of inflammatory factors is mainly concentrated in vascular smooth muscle cells (VSMCs).^16^ In vitro studies of smooth muscle cells (SMCs) found that the secretion of the inflammatory mediators‐known as matrix metalloproteinases (MMPs) could be induced by high wall‐shear stress (WSS).^17^ Phenotypic modulation occurs in VSMCs and refers to the transformation of VSMCs from a contractile phenotype to a pro‐inflammatory phenotype, which is characterized by the decreased expression of α‐SMA and SM22α and increased expression of MMPs. To fully understand this process, it is necessary to define the mechanisms behind VSMC phenotypic modulation. Numerous cytokines have been reported to be involved in VSMC phenotypic modulation,^18^, ^19^, ^20^ of which tumor necrosis factor‐alpha (TNF‐α) is critical to IA pathology.^20^

Epidermal growth factor receptor (EGFR) signaling is versatile and has been implicated in multiple vascular diseases,^21^, ^22^ and all members of the EGFR family and most EGFR ligands are expressed in VSMCs.^21^ In a study of SMC phenotypes in primary culture, EGF, heparin‐binding EGF‐like growth factor (HB‐EGF), and epiregulin (ER) were found to bind to EGFR and stimulate phenotypic modulation of contractile VSMCs. EGFR is not only activated by common EGFR ligands but also by factors including angiotensin II (Ang II), norepinephrine, and TNF‐α; this phenomenon of non‐typical activation is called “transactivation.” Of these factors, TNF‐α has been reported to induce EGFR activation in human tracheal SMCs.^23^

Epidermal growth factor receptor activation‐induced VSMC phenotypic modulation is mediated by the downstream effectors extracellular signal‐regulated kinase 1/2 (ERK1/2) and p38 mitogen‐activated protein kinase (p38 MAPK).^24^ Erlotinib is a selective inhibitor of EGFR that reversibly binds to the ATP binding site of EGFR, blocking the trans‐phosphorylation of its homodimer.^25^ Although erlotinib has recently been reported to protect against abdominal aortic aneurysm,^22^ the role of EGFR in IA formation remains arcane. In the present study, we identified the expression level of phosphorylated EGFR in human IA samples and used erlotinib to validate the biological role of EGFR in a model of model of IA. In addition, we explored the activation of EGFR and downstream signaling pathways in WSS‐stimulated VSMCs.

## 2 MATERIALS AND METHODS

### 2.1 Human samples

The IA and superficial temporal artery tissue samples were collected during neurosurgical clipping surgery of intracranial aneurysms and arteriovenous malformation surgery at the Department of Neurosurgery, Changhai Hospital, Second Military Medical University, Shanghai, China. We obtained informed consent from patients for their participation in the study. The study was approved by the Operative Ethical review committee of Second Military Medical University Changhai Hospital and approval number is CHEC2017‐073.

### 2.2 Animals

All rats were purchased and raised at the Experimental Animal Center of Changhai Hospital, Second Military Medical University. All procedures with animals were in accordance with guidelines on the use and care of laboratory animals for biomedical research published by National Institutes of Health (No. 85‐23, revised 1996), and the experimental protocol was reviewed and approved by the ethical committees of Second Military Medical University. Moreover, all animal data reporting has followed the ARRIVE guidelines.^26^ Animals *w*ere randomized, and two investigators were blinded to group allocation during data collection. At least three repetitions of independent samples were analyzed in all experiments.

### 2.3 Cerebral aneurysm induction surgery

Male Sprague‐Dawley rats (8 weeks old, 180–200 g) were used. The rats were housed individually and kept on a 12‐h light/dark cycle at 25°C. As previously reported, rats were anesthetized by intraperitoneal injection of pentobarbital sodium at 40 mg/kg body weight. Rats were randomly divided to three groups (*n* = 30 per group): sham, IA, and IA + Erlotinib group. The number of animals was determined by the need of following assays.

The right common carotid artery, left external carotid artery, and left pterygopalatine artery were ligated to establish intracranial aneurysm model for rats in IA and IA + Erlotinib group. Sham operation was performed for rats in control group.^27^ After IA induction, rats in IA group were fed the same as the rats in control group. Erlotinib (3.75 mg/kg/day, dissolved in 6% Captisol) was intraperitoneal injected for rats in Erlotinib group. The same dose of 6% Captisol was injected for rats in vehicle group. Erlotinib was purchased from MedChemExpress (Catalog NO. HY‐12008).

### 2.4 Vessel casting

After 3 months, rats in all groups (*n* = 10 rats per group) were anesthetized by intraperitoneal as previous mentioned, then perfused through heart using normal saline then casting agents (Batson's #17). After 24 h, rat brains with vascular cast were removed from cranial bones. After immersed in 10% potassium hydroxide solution for another 24 h, brain tissues and vessel walls were eroded with vessel cast left. Scanning electron microscopy (S4800; Hitachi) was applied to observe the vessel cast. The severity of IA was classified into four grades according to electron microscopic pathological changes. Normal vessel was identified as Grade 0; the IA was Grade 1 when anterior communicating artery (AComA) was tortuous regularly; Grade 2 when AComA was tortuous irregularly; Grade 3 when saccular aneurysm was formed (Figure 4B).

### 2.5 Cell culture

Rat brain VSMCs and endothelial cells (ECs) were separated from the Willis circle of 10 weeks old male SD rats. The brain vessels were harvested under sterile conditions, washed by PBS, and removed excess fat and fascia from adventitia and attached wall blood cells. After that, the vessels were placed in enzyme solution containing 0.2% collagenase II and 1 mg/ml soybean trypsin inhibitor for 20 min at 37°C. The digested ECs were collected by centrifuge, and the supernatant was discarded. The collected ECs were suspended by DMEM media (10% FBS, 1% P/S) and cultured in a humidified atmosphere of 95% air and 5% CO_2_ at 37°C. Brain ECs were cultured and observed under phase contrast microscope: polygonal cells showed monolayer, paving stone mosaic or petal‐like shape.

For VSMCs isolation, the blood vessels were cut off along the long axis and intima was tore off. Then, the vessels were cut it into 1 mm^3^ pieces and placed in fourfold volume of 0.125% trypsin for 15 min at 37°C. DMEM containing 10% FBS was added to stop digestion. The digested VSMCs were collected by centrifuge, and the supernatant was discarded. The collected cells were grown in culture bottles with DMEM containing 20% FBS. After 3 days of primary culture, the cells grew adhering to the wall. After 2 weeks, the cells were observed to be spindle‐shaped. Cells were changed into Smooth Muscle Cell Media (ScienCell) after passage 2‐3.

Human brain vascular smooth muscle cells were purchased from ScienCell (Catalog NO. 1100). The cells were resuscitated in Smooth Muscle Cell Media (ScienCell) and used for experiment after 3–5 times of passage. Recombinant human TNF‐α protein was purchased from Abcam (Catalog NO. ab259410). TNF‐α neutralizing antibody was purchased from Sino Biological (Catalog NO. 10602‐R10N1).

### 2.6 EC‐VSMC co‐culture model

Primary ECs and VSMCs were inoculated on both sides of PET track‐etched membrane (FALCON 353090), respectively. After the cells grew well, they were taken out under aseptic conditions and placed in Parallel‐Plate Flow Chamber (Figure 5A). The experiment was carried out at 37°C, and the perfusate was DMEM containing 10% FBS. The WSS applied on the ECs was regulated by controlling the flow rate of the circulating device. The WSS in this experiment was shown as grouping: the action time concluding 24, 48, and 72 h; the WSS concluding 0, 4, 12, and 36 dyn/cm^2^. The cells used in this assay were between passages 3 and 6.

### 2.7 Quantitative real‐time polymerase chain reaction analysis

At the time of harvest, rats in all groups (*n* = 6 rats per group) were anesthetized by intraperitoneal as previous mentioned. The vessels from Willis circle of rats were collected and kept in RNAlater reagent (Thermo Fisher AM7020). Total RNA of vessels was extracted by TRIzol Reagent (Ambion) and reversed‐transcribed using the S PrimeScript™ RT reagent Kit (TAKARA). Real‐time PCR was performed on the LightCycler^®^ 96 (Roche) machine using SYBR^®^ Premix Ex Taq™ II (Tli RNaseH Plus) (TAKARA). Primer sequences were summarized in Table 1. The expression of genes relative to 18S was determined by 2^−ΔΔ^ *^C^* ^t^ method.

**TABLE 1:** Primer sequences of RT‐qPCR in this paper

Gene name	Forward Primer	Reverse Primer
h‐SM α‐actin	AATGCAGAAGGAGATCACGG	TCCTGTTTGCTGATCCACATC
h‐Calponin	AACCATACACAGGTGCAGTC	GATGTTCCGCCCTTCTCTTAG
h‐SM22α	TCCAGACTGTTGACCTCTTTG	TCTTATGCTCCTGCGCTTTC
h‐ TNF‐α	GAGCACTGAAAGCATGATCC	CGAGAAGATGATCTGACTGCC
h‐GAPDH	GATGCCCCCATGTTCGTCAT	TCTTCTGGGTGGCAGTGATG
r‐ TNF‐α	CTGTGCCTCAGCCTCTTCTC	ACTGATGAGAGGGAGCCCAT

### 2.8 Western blot

At the harvest time, rats in all groups (*n* = 6 rats per group) were anesthetized by intraperitoneal as previous mentioned. The vessels of Willis circle from surgical rats were collected, and total protein was extracted. Proteins from different groups were electrophoresed through 8% SDS‐PAGE gel, transferred to PVDF membrane, and immunoblotted with antibodies against EGFR (Cell Signaling, 2232); P‐EGFR (Tyr1068, Cell Signaling, 3777), GAPDH (Cell Signaling, 5174), MMP‐2(Abcam, ab37150), p38 MAPK (Cell Signaling, 8690), p‐p38 MAPK (Cell signaling, 4511), p‐Erk1/2 (Cell signaling, 4370), Erk1/2 (Cell signaling, 4695), and β‐actin (Abcam, ab8224). Immunoreactive bands were visualized by enhanced chemiluminescence. Protein bands were quantified by densitometry using ImageJ software.

### 2.9 IHC, IF and HE staining and EVG staining

At 3 months, rats in all groups (*n* = 6 rats per group) were anesthetized by intraperitoneal as previous mentioned. Rats were sacrificed and perfused with phosphate‐buffered saline followed by perfusion with 4% paraformaldehyde dissolved in PBS. Then, the brain was removed and fixed in 4% paraformaldehyde for 24 h. Immunohistochemistry (IHC) was performed as previously described.^28^ The primary antibodies for against the following proteins were used: P‐EGFR (Cell Signaling, 3777), MMP‐2 (Abcam, ab2462), and MMP‐9 (Abcam, ab86657), α‐SMA (Abcam, ab5694). All slides were observed at 20× or 40×, and all images were acquired using an Olympus DP80 microscope.

#### 2.9.1 Immunofluorescence

After rat brain was fixed in paraformaldehyde, the paraffin section was made as previously described.^25^ Then, the paraffin section was baked for 15 min. Treat the sections with xylene and alcohol. Antigen retrieval: use citrate buffer solution (pH 6.0) repair sections for 10 min with high pressure, then natural cooling to room temperature. Wash sections by PBS for 3 times × 5 min. Transmembrane by heat: penetrate section by 3% TritionX‐100 for 30 min and wash section by PBS for 3 times for 5 min. Block section by 3% BSA for 30 min. Wash section by PBS for 3 times × 5 min. Incubate with primary antibody: dilute the primary antibody by antibody diluent and incubated overnight at 4°C. Wash section by PBS for 3 times × 5 min. Incubate with fluorescent second antibody: dilute antibody by PBS at 1:300 and incubate at 37°C for 30 min in dark. Wash section by PBS for 3 times × 5 min. Stain section by DAPI at room temperature for 2 min in dark. Wash section by PBS for 3 times × 5 min. Seal section by glycerol. Photographs were taken using Olympus DP80 fluorescence microscope. The primary antibodies for the following proteins were used: p‐EGFR (Cell Signaling, 3777), MMP‐2 (Abcam, ab2462), α‐SMA (Abcam, ab5694), and TNF‐α (Proteintech, 17590‐1‐AP). The fluorescent second antibodies (Invitrogen, A21206 and A21203) were used.

#### 2.9.2 Hematoxylin‐eosin staining

The paraffin slices from the paraffin blocks prepared in IHC were dried at 60°C, and then dipped in a xylene washer twice for 5 min each time followed by anhydrous ethanol and 95% ethanol for about 1 min, respectively. The slides were soaked in 80% ethanol for 1 min and tap water for 2 min and dyed with hematoxylin dye for 3–5 min and tap water for 1–2 min. The blued sections were colored with ethanol hydrochloride solution (95% ethanol solution containing 1% HCl) and immersed for 2 s. Then, the sections were blued in tap water immediately. The slices were rinsed with weak tap water for 25 min. After the slides were dehydrated twice with 95% ethanol for about 10 s each time, spited twice with absolute ethanol for 1–2 min each time and cleared xylene for 1–2 min each time. Finally used sealing gel to adhesive tape and then observed it with microscope.

#### 2.9.3 EVG staining

The paraffin sections were dewaxed in distilled water and then incubated for 30 min at room temperature with Verhoeff's hematoxylin. Then rinsed slides lightly with tap water, differentiated slides by 2% ferric chloride solution until the gray background of black fiber appeared. Washed slides with distilled water. 5% sodium thiosulfate was used to remove iodine from slides for 1 min. Washed slides with distilled water again. Van Gieson's solution was repeated for 5 min. Dehydrated slides by absolute alcohol. After treating by xylene, the slides were sealed with neutral gum, dried, and stored.

### 2.10 Statistical analysis

GraphPad Prism for Windows (version 5.0) was used to conduct the statistical analyses. All data were shown as Mean ± SD. Student's *t* test was used to compare the statistical significance of two independent groups. Kruskal‐Wallis H test was used to analyze the incidence of IA formation, and Nemenyi test was used to compare the difference of IA formation between each group. The data normality was checked by Kolmogorov‐Smirnov test.

## 3 RESULTS

### 3.1 EGFR is activated in human IA and VSMCs undergo phenotypic modulation in human IA

**FIGURE 1:**
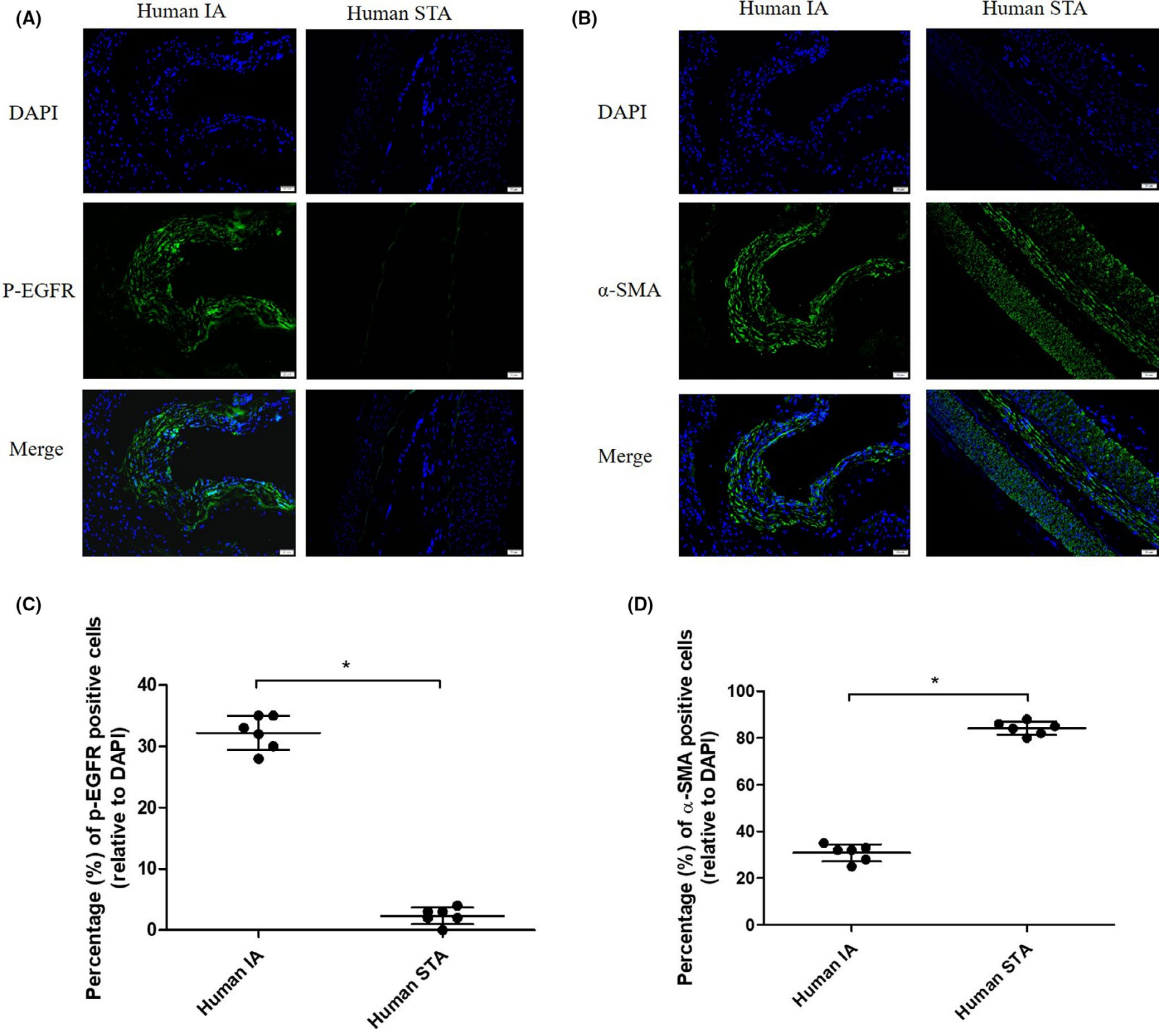
p‐EGFR (Tyr1068) and α‐SMA distribution in human intracranial aneurysm (IA) and superficial temporal artery (STA). (A,B) Representative images were shown. STA and IA were labeled with antibodies against α‐SMA (green) and p‐EGFR (green) and stained with DAPI (blue). (C,D) Quantitative analysis of cells positive for p‐EGFR and α‐SMA. *p < 0.05 vs. STA. EGFR, epidermal growth factor receptor [Colour figure can be viewed at wileyonlinelibrary.com]

We first investigated the role of phosphorylated EGFR (p‐EGFR) in IA and used immunofluorescence (IF) to assess the abundance of p‐EGFR and α‐SMA in human IA samples and superficial temporal artery (STA) samples from bypass surgeries. As shown in Figure 1A, the expression of p‐EGFR was distributed in most intracranial aneurysm wall layers. Compared to STAs, the expression of p‐EGFR significantly increased in IA walls (p‐EGFR positive cells, 2.33% vs. 32.66%, respectively, *p* < 0.05; Figure 1C). Expression of α‐SMA (Figure 1B) was downregulated in IA walls compared to STAs (α‐SMA positive cells, 84% vs. 31.66%, respectively, *p* < 0.05; Figure 1D). We also analyzed the expression for phenotype markers of VSMC, (α‐SMA and MMP‐2 in human IA walls and STAs) by immunohistochemistry (IHC) (Figure S1), and our results showed that MMP‐2 expression was upregulated in IA whereas that of α‐SMA was downregulated, suggesting phenotypic modulation of VSMCs in IA. TNF‐α, another pro‐inflammatory cytokine, was also found to be induced in IA walls (Figure S1).

### 3.2 EGFR is activated in cerebral vessels of the IA rat model

The development of IA is associated with abnormal hemodynamics and arterial remodeling. We previously performed microarray analysis to explore mRNA expression profiles of VSMCs in the IA rabbit model induced by high flow at day 7.^28^ These data that were deposited in the NCBI's Gene Expression Omnibus (GSE61212) revealed that some EGFR ligands (HBEGF, EGF domain‐specific O‐linked N‐acetylglucosamine transferase [EOG]T, EGF fibronectin type III, laminin G domains [EGFLAM], and epiregulin [EREG]) were upregulated in rabbit VSMCs under hemodynamic stimuli (Table 2) indicating that the EGFR signaling pathway participates in the initiation of IA.

**TABLE 2:** Gene expression of EGFR ligands in the rabbit IA model (data from GSE61212)

Gene name	Fold change	*p*‐Value
HBEGF	3.086729893	0.002202393
EOGT	2.195224001	0.043278565
EGFLAM	2.372878003	0.042389307
EREG	6.328621013	0.003798676

**FIGURE 2:**
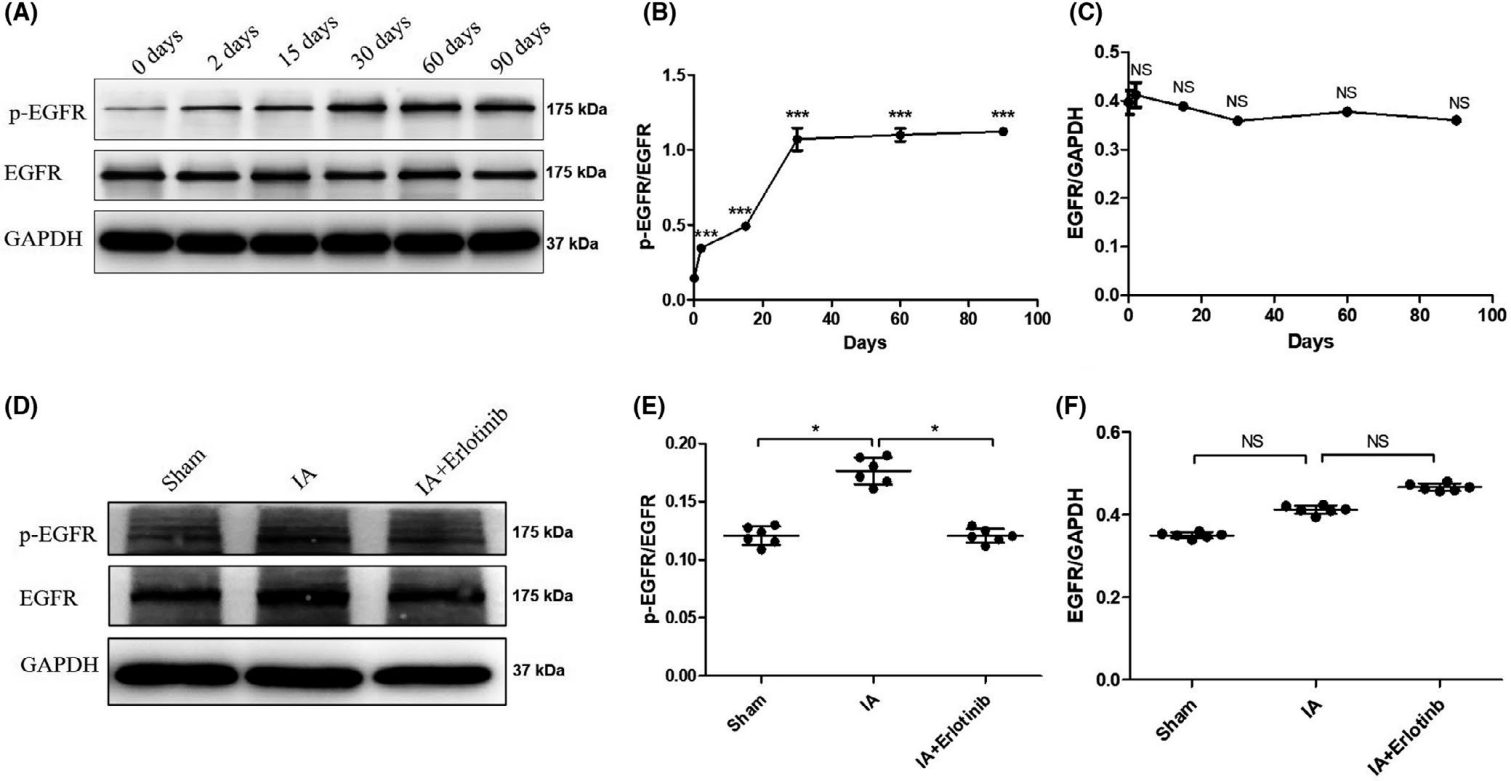
EGFR phosphorylation in the brain vessels of Willis circle of rat intracranial aneurysm (IA) models. (A) At days 2, 15, and 30, the arteries were taken for Western blot analysis of p‐EGFR and total EGFR. (B) Expression of p‐EGFR was quantified by densitometry and normalized by expression of EGFR. ***p < 0.001 vs. 0 Day. (C) Expression of EGFR was quantified by densitometry and normalized by expression of GAPDH. ^NS^ *p* > 0.05 vs. 0 Day. (D) With the treatment of Erlotinib for 30 days, the arteries were taken for p‐EGFR and EGFR by Western blot. (E and F) The expression levels of p‐EGFR and EGFR were quantified and normalized. ^NS^ *p* > 0.05; *p ≤ 0.05. Data are mean ± SD, *n* = 6. EGFR, epidermal growth factor receptor

To investigate EGFR signaling in the development of IA, a rat IA model was induced by ligation of the unilateral common carotid artery, and contralateral pterygopalatine and external carotid arteries. As shown in Figure 2A, arterial ligation induced EGFR activation as assessed by Tyr1068 phosphorylation of EGFR in the rat Circle of Willis (the circular anastomosis of cerebral vessels) on days 2, 15, 30, 60, and 90. EGFR activation reached its peak on day 30; and the ratio of p‐EGFR to total EGFR increased over time (Figure 2B), whereas total EGFR expression levels were consistent over the 90‐day analysis period (Figure 2C). These data revealed that increased EGFR activation was derived from the elevation of p‐EGFR but not from total EGFR. In contrast to the controls, rats treated with erlotinib exhibited decreased p‐EGFR on day 30 (Figure 2D, and quantitative‐analysis indicated a 30% reduction in p‐EGFR in erlotinib‐treated rats (Figure 2E). Further analysis indicated that the change in p‐EGFR derived from the p‐EGFR itself (Figure 2F).

### 3.3 Pharmacological inhibition of EGFR protects against vascular remodeling in rats

**FIGURE 3:**
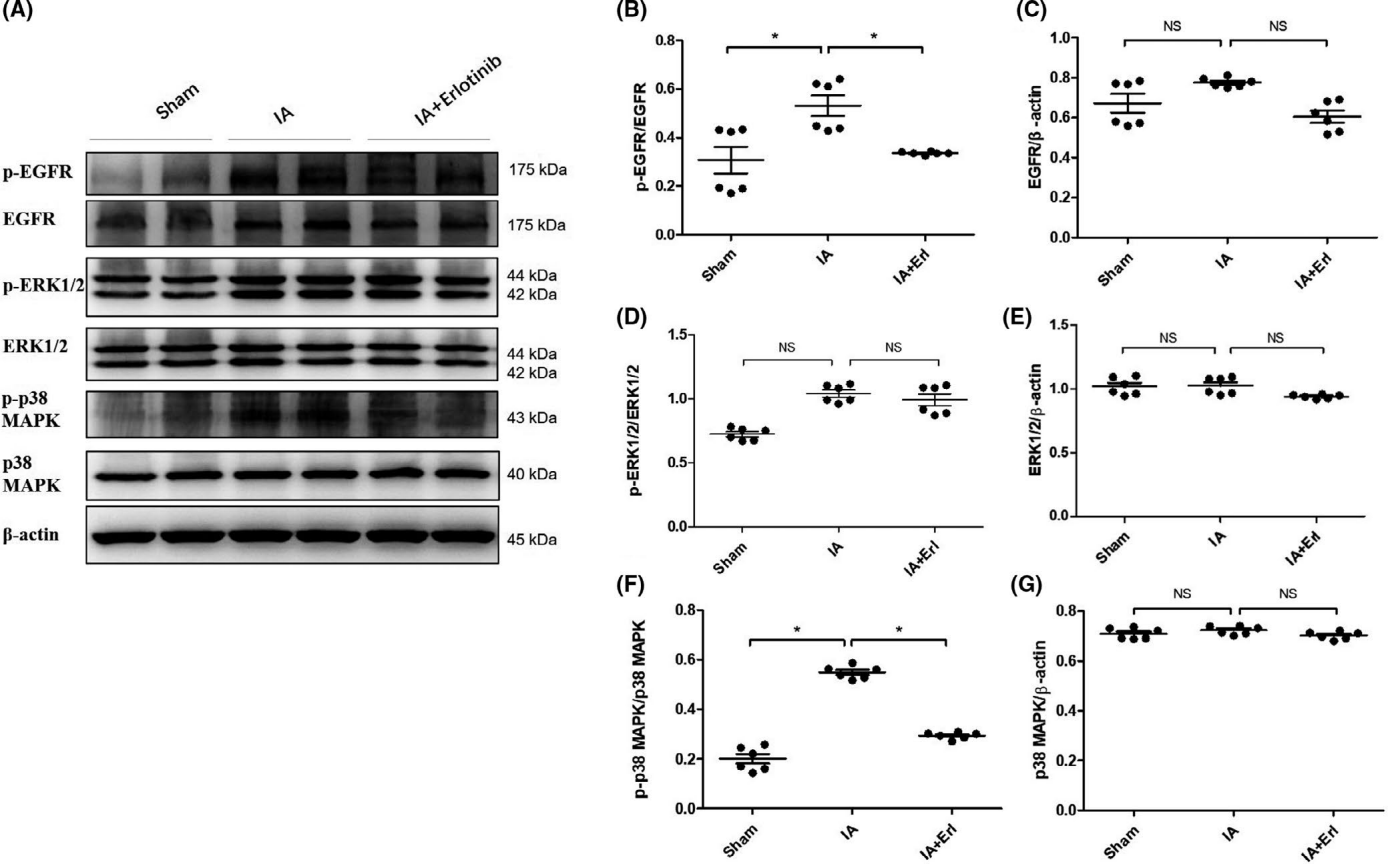
EGFR activation and downstream signals in the cerebral arteries of rat intracranial aneurysm (IA) models with the treatment of erlotinib. (A) At months 3, the arteries were taken for analysis of p‐EGFR, EGFR, p‐Erk1/2, Erk1/2, p‐p38MAPK, and p38MAPK. (B, D and F) The expression levels of p‐EGFR, p‐Erk1/2, and p‐p38MAPK were quantified and normalized by their unphosphorylated forms. ^NS^ *p* > 0.05; *p ≤ 0.05. (C) (E and G) The expression levels of EGFR, Erk1/2, and p38MAPK were quantified and normalized by β‐actin. ^NS^ *p* > 0.05. Data are represented as the mean ± SD, *n* = 6. EGFR, epidermal growth factor receptor

To evaluate the role of p‐EGFR in IA development and to examine whether EGFR can be therapeutically targeted for IA treatment, we induced IA in rats and treated the rats simultaneously with an intraperitoneal injection of erlotinib at a dosage of 3.5 mg/kg/day, which is congruent with a previous report.^29^

p‐EGFR (Tyr1068) expression in the rat Circle of Willis was evaluated by immunoblotting 3 months after arterial ligation; and as shown in Figure 3A, p‐EGFR expression was highest in the IA group, whereas p‐EGFR expression decreased in the erlotinib‐treated group. Analysis of the ratios of p‐EGFR to total EGFR and EGFR to β‐actin (Figure 3B,C) displayed a reduction in p‐EGFR levels.

**FIGURE 4:**
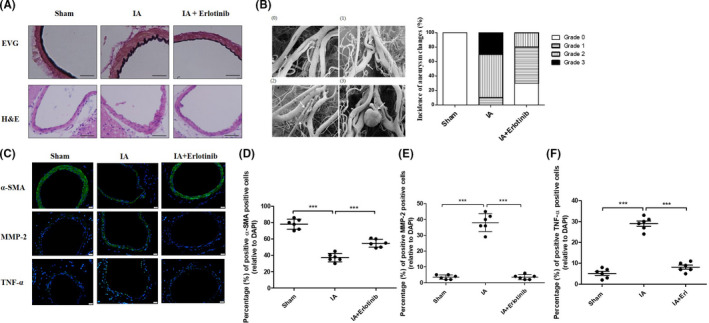
Erlotinib reduced incidence of intracranial aneurysm (IA) and phenotype modulation of vascular smooth muscle cells (VSMCs) at months 3 of rat IA models. (A) At months 3, the remodeling of vascular walls in hemodynamic stress‐induced IA was analyzed for their histological features by EVG (upper, elastic fibers: black, collagen fibers: red) and H&E (lower) staining. Representative images were shown. Scale bars, 50 μm (B) Left: representative images of the severity of IA according to electron microscopic pathological changes. (0) Grade 0; (1) Grade 1; (2) Grade 2; (3) Grade 3. Right: statistical analysis of the severity of IA formation for each group after 3 months induction. *N* = 10 for sham group; *n* = 10 for IA group; *n* = 10 for erlotinib group. (C) Tissue sections of the vascular walls were taken for analysis of p‐EGFR, α‐SMA, MMP‐2 and TNF‐α by IF (green: protein; blue: DAPI). Scale bars, 20 μm. (D, E and F) Quantitative analysis of cells positive for α‐SMA, MMP‐2, and TNF‐α. ***p ≤ 0.001 vs. IA. EGFR, epidermal growth factor receptor [Colour figure can be viewed at wileyonlinelibrary.com]

Destructive vascular remodeling plays an important role in IA formation.^30^ Use of Verhoeff Van Gieson (EVG) stain in sections of the anterior cerebral artery (ACA) of rat IA models signaled that hemodynamic stress caused thinning of the media layer and disruption of the elastin fibers in vascular walls. Notably, jagged folds and disruption of elastin fibers were less pronounced in the erlotinib‐treated group (Figure 4A). Additionally, Hematoxylin‐eosin (H&E) staining of ACA sections confirmed the reduction in vascular remodeling in the erlotinib‐treated group as shown in Figure 4A. Collectively, these results demonstrated that erlotinib reduced vascular remodeling.

Ten rats from each group (sham, IA, and IA + erlotinib) were examined for the incidence of aneurysms according to four grades, and representative images of each grade obtained by electron microscopy are shown in Figure 4B. Rats in the sham group showed no signs of aneurysm; in the IA group, 10% exhibited grade 1 aneurysms, 60% grade 2, and 30% grade 3 (Figure 4B); and in the Erlotinib‐treated group, 30% showed signs of normal vessels, 50% exhibited grade 1 aneurysms, and 20% exhibited grade 2. The incidence of IA was lower in the erlotinib‐treated group relative to the IA group (*p* = 0.0118) (Table S1).

### 3.4 Pharmacological inhibition of EGFR attenuates IA model induced the suppression of α‐SMA and increment in MMP‐2 in VSMCs

From the current data, EGFR activation appeared to be critical to IA development. However, whether EGFR activation directly induced the modulation of VSMC phenotype in the IA formation remained to be determined. To investigate this, further, we examined the expression of phenotypic markers in rats from the sham group, the IA group, and the IA + erlotinib group at 3 months using immunofluorescence (IF). As shown in Figure 4C,D, α‐SMA principally distributed in the media layer was attenuated in the IA group and nearly abrogated in the erlotinib‐treated group. MMP‐2 and TNF‐α were also detected simultaneously, and our results revealed that their expression levels increased in the IA group, whereas the increment was attenuated in the IA + erlotinib group (Figure 4E,F). TNF‐α was expressed in both the media and endothelial layers of the vessel wall. These findings suggested that changes materialized in the VSMC phenotypic marker proteins α‐SMA and MMP‐2 after IA induction and that these changes could be attenuated by treatment with the EGFR inhibitor erlotinib.

### 3.5 IA model‐induced EGFR activation is required for the activation of p38 MAPK and Erk1/2

It has been reported that downstream effectors of EGFR (including ERK1/2 and p38 MAPK) activate VSMC phenotypic modulation.^31^, ^32^ However, the molecular mechanisms underlying VSMC phenotypic modulation during artery ligation‐based IA model induction remain unclear. To investigate the underlying signaling pathways contributing to VSMC phenotypic modulation, we determined the expression levels of ERK1/2 and p38 MAPK in the Circle of Willis in rats of the sham group, the IA group, and the IA + erlotinib group. As shown in Figure 3D‐G, the IA group showed an increase in the expression of phosphorylated p38 MAPK and ERK1/2 compared to the sham group, but the expression levels of total p38 MAPK and ERK1/2 remained consistent among different groups. We observed that erlotinib treatment significantly reduced the expression of phosphorylated p38 MAPK, suggesting that EGFR activation was required for p38 MAPK activation (Figure 3A,F,G). Erlotinib treatment also slightly reduced the expression of phosphorylated ERK1/2. Taken together, these data suggested that IA model‐induced EGFR activation was critical for the activation of downstream p38 MAPK, which then led phenotypic modulation of VSMC. ERK1/2 activation may also participate in this process.

### 3.6 WSS‐induced EGFR activation is required for VSMC phenotypic modulation in vitro

**FIGURE 5:**
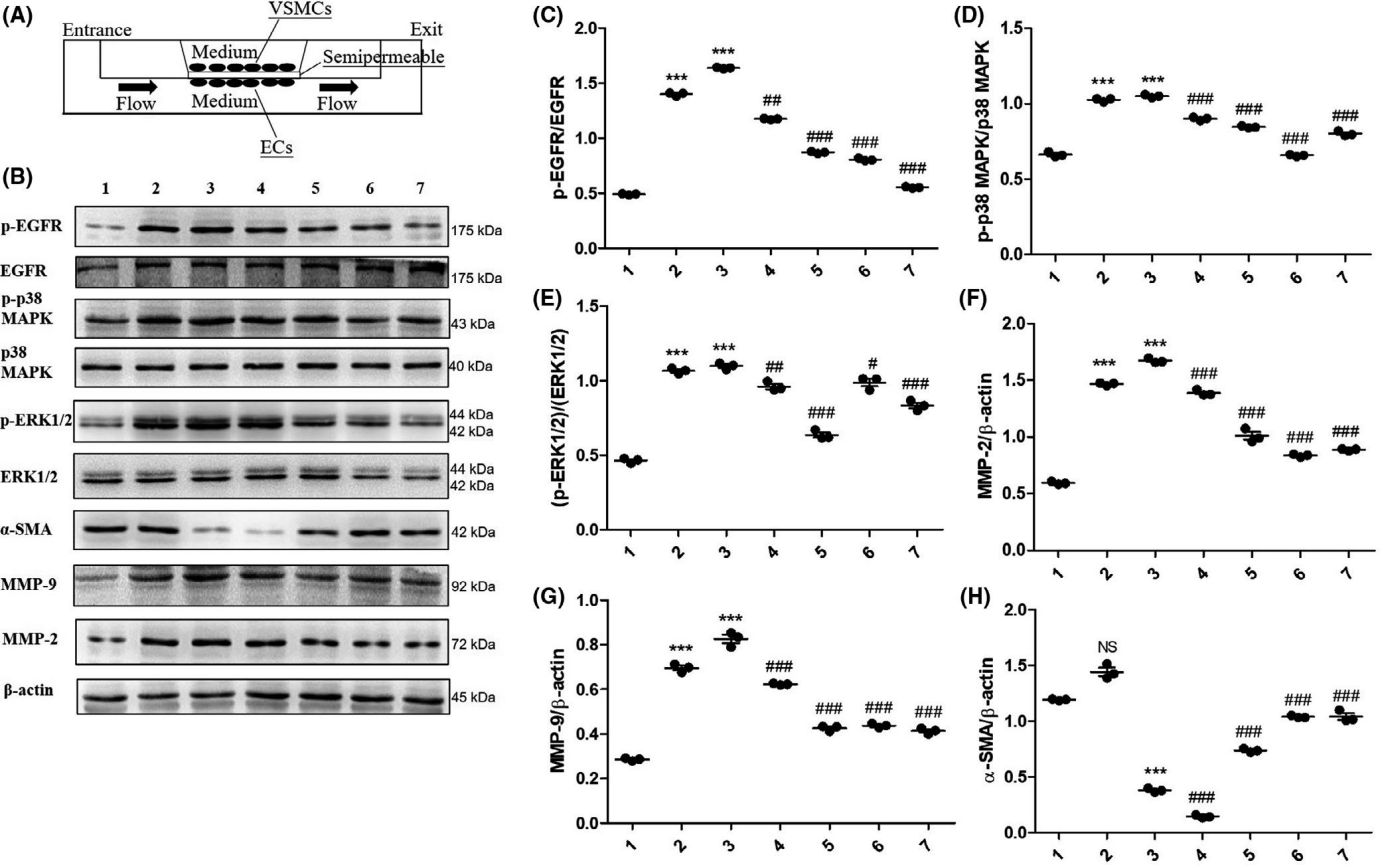
Erlotinib reduced phenotype modulation of vascular smooth muscle cell (VSMC) induced by hemodynamics in in vitro co‐culture model. (A) Co‐culture model of VSMCs and endothelial cells (ECs). (B) Expression levels of p‐EGFR, EGFR, p‐Erk1/2, Erk1/2, p‐p38 MAPK, p38 MAPK, α‐SMA, MMP‐2, and MMP‐9 under the conditions of wall‐shear stress (WSS) stimulation with or without different concentrations of Erlotinib (1: 0 dyn/cm^2^; 2: 30 dyn/cm^2^; 3: 36 dyn/cm^2^; 4: 36 dyn/cm^2^ + 1 nM erlotinib; 5: 36 dyn/cm^2^ + 5 nM erlotinib; 6: 36 dyn/cm^2^ + 25 nM erlotinib; 7: 36 dyn/cm^2^ + 125 nM erlotinib). (C, D and E) In each group, the expression levels of p‐EGFR, p‐Erk1/2, and p‐p38MAPK were quantified and normalized by their unphosphorylated forms. ***p ≤ 0.001 vs. 0 dyn/cm^2^. ^###^ *p* ≤ 0.001 vs. 36 dyn/cm^2^. (F, G and H) In each group, the expression of α‐SMA, MMP‐2, and MMP‐9 was quantified and normalized by β‐actin. Data are represented as the mean ± SD, *n* = 3. **p ≤ 0.01; ***p ≤ 0.001 vs. 0 dyn/cm^2^. ^#^ *p* ≤ 0.05; ^##^ *p* ≤ 0.01; ^###^ *p* ≤ 0.001 vs. 36 dyn/cm^2^. EGFR, epidermal growth factor receptor

The intracranial vessel wall is composed of multiple structures, including the adventitia, the media layer, and the tunica intima. ECs are present in the intima layer, which is directly exposed to shear stress as a result of blood flow. VSMCs are the most important components of the medial layer, which endure blood flow stress indirectly. To reproduce the structure of the intracranial arterial vascular wall, we established an in vitro co‐culture model of ECs and VSMCs extracted from rat ACAs to validate EGFR activation induced by hemodynamic stress as previously reported.^33^ As shown in Figure 5A, VSMCs and ECs were co‐cultured on opposite sides of one pellicle before being placed in a flow chamber, and VSMCs were cultured in resting media, whereas ECs were stimulated by WSS at different strengths and stimulation times in flowing media.

To investigate the appropriate stimulatory duration and strength for WSS in the co‐culture model, experiments were conducted under the conditions listed in Figure S2. At the end of the experiment, VSMCs were collected and evaluated for the expression of EGFR, p‐EGFR (Y1068), α‐SMA, MMP‐2, and MMP‐9 by immunoblotting. As shown in Figure S2A, the expression levels of p‐EGFR increased concomitantly with increasing WSS strength and stimulation duration and reached a peak when the strength of WSS was 36 dyn/cm^2^ and the length of stimulation was 24 h; the expression levels of total EGFR, however, remained consistent. The analysis shown in Figure S2C demonstrated that elevation of p‐EGFR originated from p‐EGFR itself and not from total EGFR. The expression levels of α‐SMA diminished commensurately with increasing strength of WSS and extension of stimulation duration, whereas the expression levels of MMP‐2 and MMP‐9 were augmented. These results indicated that VSMCs were modulated to a pro‐inflammatory phenotype from a contractile phenotype as a result of WSS strength and stimulation duration.

To validate the inhibitory effects of erlotinib on phenotypic modulation of VSMCs, we used differing concentrations of erlotinib (1, 5, 25, 125 nM) in the medium for 24 h at a WSS of 36 dyn/cm^2^. As shown in Figure 5B,C, expression of p‐EGFR was inversely correlated with increasing concentrations of erlotinib, whereas the expression of total EGFR remained constant. Moreover, as shown in Figure 5F‐H, α‐SMA increased and MMPs decreased with reduced expression of p‐EGFR. Importantly, treatment of erlotinib at a concentration of 25 nM completely reversed the VSMC phenotypic modulation induced by high WSS (36 dyn/cm^2^). These data revealed that EGFR activation was required for VSMC phenotypic modulation induced by WSS in vitro.

### 3.7 WSS‐induced EGFR activation is required for the activation of p38 MAPK and ERK1/2 in vitro

Following the exploration of p38 MAPK and ERK1/2 activation in IA rats, we next sought to validate their significance in vitro. Using our previously established co‐culture cell model, the expression levels of p‐p38 MAPK and p‐ERK1/2 in VSMCs were assessed by immunoblotting, and Figure S2 illustrates that the changes in p‐p38 MAPK and p‐ERK1/2 were positively correlated with p‐EGFR as a consequence of increasing WSS strength and stimulation time, while total p38 MAPK and ERK1/2 remained steady. As shown in Figure 5B, erlotinib treatment curtailed the expression of p‐p38 MAPK and p‐ERK1/2, and additional analysis implied that the incremental increases in p‐p38 MAPK and p‐ERK1/2 as well as their attenuation following erlotinib treatment were not from total p38 MAPK and ERK1/2 (Figure 5D,E). We demonstrated that WSS induced the observed VSMC phenotypic modulation via p38 MAPK and ERK1/2 and that this induction was attenuated by the p‐EGFR inhibitor erlotinib.

### 3.8 EGFR mediates TNF‐α induced VSMC phenotypic modulation

**FIGURE 6:**
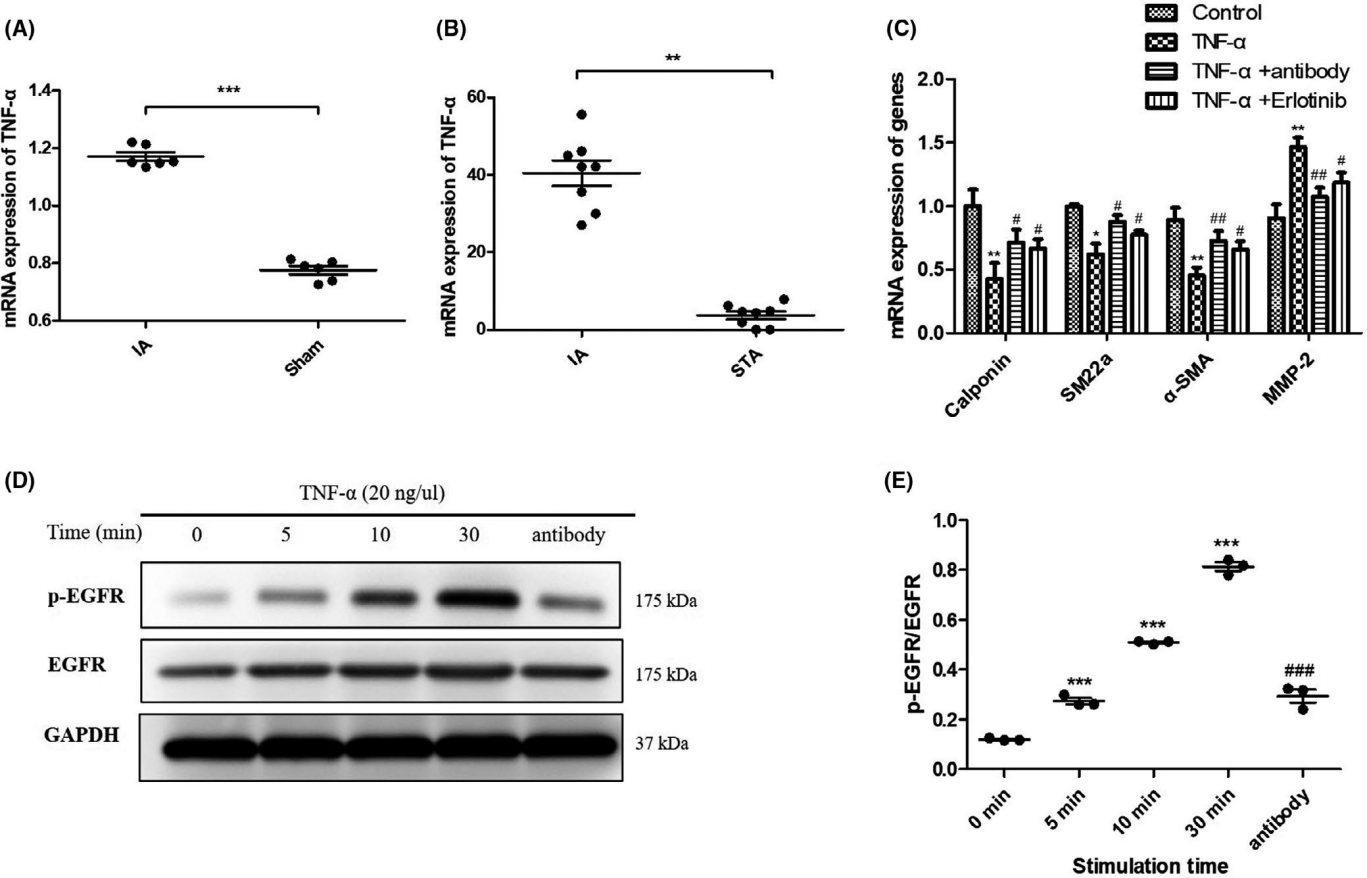
TNF‐α transactivated EGFR to induce phenotypic modulation of vascular smooth muscle cells (VSMCs). (A) mRNA expression level of TNF‐α in intracranial aneurysm (IA) rat model and sham group analyzed by qPCR. ***p ≤ 0.001. (B) mRNA expression level of TNF‐α in unruptured human IA and superficial temporal artery (STA) samples analyzed by qPCR. **p ≤ 0.01. (C) mRNA expression level of SM22α, α‐SMA, Calponin, and MMP‐2 in VSMCs with stimulation of TNF‐α (20 ng/µl), TNF‐α (20 ng/µl) + antibody (1 µg/µl), and TNF‐α (20 ng/µl) + erlotinib (100 nM) analyzed by qPCR. *p ≤ 0.05; **p ≤ 0.01. (D) Protein expression of p‐EGFR, EGFR in VSMCs stimulated by TNF‐α (20 ng/µl) for different time analyzed by Western blot. (E) The expression levels of p‐EGFR were quantified and normalized by EGFR. Data are represented as the mean ± SD, *n* = 3. ***p ≤ 0.01 vs. 0 min, ^###^ *p* ≤ 0.001 vs. 30 min. EGFR, epidermal growth factor receptor

TNF‐α has been shown to induce VSMC phenotypic modulation in IA pathology.^20^ In animal experiments, IF staining revealed that TNF‐α was activated in the IA group (Figure 4C) and that TNF‐α mRNA expression levels in the IA group as determined by qPCR in ACA tissues increased compared to the sham group (Figure 6A). We also detected the mRNA levels for TNF‐α in human samples, and as shown in Figure 6B, the TNF‐α expression in human IA tissues was significantly higher than that in STA tissues. We preliminarily explored whether TNF‐α was involved in the process of EGFR‐induced VSMC phenotypic modulation in vitro and showed that stimulation of human brain vascular smooth muscle cells (HBVSMCs) with TNF‐α (20 ng/µl) reduced mRNA expression of α‐SMA, calponin, and SM22α compared to the untreated control group (Figure 6C). TNF‐α stimulation also elevated MMP‐2 mRNA expression. These results validated the previous finding that TNF‐α could induce VSMC phenotypic modulation. HBVSMCs were additionally stimulated by TNF‐α (20 ng/µl) + TNF‐α neutralizing antibody (1 µg/µl) and TNF‐α (20 ng/µl) + erlotinib (100 nM). Our results (Figure 6C) showed that both TNF‐α neutralizing antibody and erlotinib attenuated VSMC phenotypic modulation induced by TNF‐α. Next, to examine whether TNF‐α activated EGFR at the protein level, we treated HBVSMCs with TNF‐α (20 ng/μl) for 5, 10, and 30 min and demonstrated that TNF‐α induced the phosphorylation of EGFR in a time‐dependent manner (Figure 6D). We also assessed the change in p‐EGFR by treatment with TNF‐α neutralizing antibody (1 µg/µl) for 30 min, and results showed that TNF‐α antibody greatly attenuated TNF‐α induced phosphorylation of EGFR (Figure 6D) (the ratio of p‐EGFR to EGFR is depicted in Figure 6E). Further study confirmed that EGFR activation was augmented proportionately with increasing TNF‐α concentration, accompanied by increments in MMP‐2 and decrements in α‐SMA (Figure S3).

Collectively, these data suggested that EGFR was transactivated by TNF‐α in HBVSMCs and that EGFR appeared to be a critical mediator of TNF‐α‐induced modulation of VSMC phenotype.

## 4 DISCUSSION

In this study, we uncovered activation of EGFR in the vascular walls of human IA. In our IA animal models, we observed that EGFR was activated in the VSMCs of cerebral vascular walls, accompanied by phenotype switching of VSMCs. Application of erlotinib attenuated the occurrence of IA, and in vitro study found that erlotinib reduced the phenotypic switching of VSMCs induced by abnormal WSS. The innovation of this study was for the first‐time observing activation of EGFR in VSMCs during IA formation and for the first time demonstrating potential use of the clinically used drug erlotinib in the treatment of IA. This study thus provides a possible direction for clinical research on noninvasive treatment of IA.

Although there are several methods used to induce IA in animals—including elastase induced aneurysms, frequent pouch aneurysms, and the use of artificial implantations—all of these methods possess their own advantages and disadvantages.^34^, ^35^ Although the elastase‐based method has been most widely used in IA research,^36^ in this study we induced rat IA models by arterial ligation due to the need for obtaining abnormal hemodynamics, and the high availability of experimental animal specimens. In IA research, it is of utmost importance to attain more information from limited samples because obtaining specimens, especially human specimens, is rather difficult. Based on this, investigators have widely exploited “omics” research and bioinformatics.^37^ In addition, the application of computers to analyze the data of aneurysm‐related fluid dynamics has also generated research interest.^38^ The precedent for our study was our previous microarray data analysis of rabbit aneurysms, in which we found that the expression of EGFR ligands increased. When we execute bioinformatics analyses in the future to analyze the previous data more deeply, we expect to produce additional novel findings.

Although previous studies on EGFR have primarily focused on other diseases such as lung cancer, there is increasing evidence to suggest that the EGFR signaling pathway occupies a crucial role in vascular diseases.^22^, ^39^, ^40^ Activation of EGFR has been reported to be involved in the formation of abdominal aortic aneurysms, and these can be prevented by inhibition of EGFR. Our results showed that p‐EGFR expression was elevated in human IA samples, and microarray analysis revealed that mRNA expression of EGFR ligands was elevated in rabbit IA models. Pharmacological blockade of EGFR using erlotinib in rat IA models disclosed that EGFR activation was required for VSMC phenotype modulation and the production of pro‐inflammatory cytokines. We also found that stimulation of ECs by WSS‐induced phenotypic modulation of co‐cultured VSMCs and that EGFR activation mediated TNF‐α‐induced phenotypic modulation in VSMCs. Our data thus suggest that EGFR serves as a key mediator regulating VSMC phenotypic modulation in IA. To our knowledge, this study is the first to show that EGFR is important in the development of IA and that it can be inhibited by an EGFR‐specific inhibitor. Our findings provide further information on the R&D of drugs for the prevention and treatment of IA.

To explore whether the activation of EGFR was transient or persisted after animal surgery, we determined the expression of p‐EGFR at different time intervals. As shown in Figure 2, p‐EGFR was induced on day 2 and increased over time, which indicated that abnormal hemodynamics could provide a constant stimulus for EGFR activation and induce VSMC transformation from a contractile phenotype to a pro‐inflammatory phenotype. Intriguingly, most of the p‐EGFR was derived from p‐EGFR itself and not from total EGFR. Previous studies on rabbit IA models showed that some EGFR ligands were upregulated just 1 week after induction surgery,^28^ and it was suggested that the activation of EGFR was associated with increased expression of EGFR ligands. However, no team has examined the ligands that are responsible for EGFR activation, which is a limitation of the current study. Therefore, additional investigation is necessary to identify the key EGFR ligand(s) involved in the development of IA. Herein, we demonstrated for the first time that EGFR activation and downstream signaling in rat cerebral VSMCs were indirectly stimulated by WSS, and we noted phosphorylation of both p38 MAPK and ERK1/2 in our rat IA models. The activation of p38 MAPK was inhibited by erlotinib, and previous work revealed that EGFR ligands triggered an SMC phenotypic via p38 MAPK^24^—further validating the present study.

Hemodynamic stress often causes destructive vascular remodeling and is characterized by disruption of the internal elastic lamina, thinning of the media, apoptosis of SMCs, and loss of fibronectin.^30^, ^41^ Workers previously showed that abnormal hemodynamics induced apoptosis of VSMCs and inhibited VSMC proliferation, leading to vascular‐wall remodeling.^42^ In our study, we found that VSMCs could be transformed to a pro‐inflammatory phenotype from a contractile phenotype with vascular‐wall remodeling. Whether this phenotypic modulation regulates VSMC proliferation remains unclear. In the current study, we found that treatment with erlotinib in animal experiments significantly reduced thickening of the vascular wall, attenuated the disruption of the internal elastic lamina, and promoted thinning of aneurysmal walls—all of which may lead to a reduced incidence of IA formation. Our animal study therefore revealed that abnormal hemodynamics consistently induced EGFR phosphorylation.

Prior work showed that shear stress stimulation induced ECs to secrete intercellular molecules so as to regulate SMC turnover.^43^, ^44^ We herein demonstrated that WSS indirectly regulated the protein expression levels in VSMCs, indicating that VSMC phenotypes were also regulated by EC‐mediated signals. This regulation was inhibited by erlotinib, indicating that EGFR activation was critical to WSS‐induced VSMC phenotypic modulation. However, we have not as yet evaluated the regulatory molecular mechanisms underlying the EC‐to‐VSMC conversion under WSS stimulation. As described previously, we found that the expression of TNF‐α was significantly increased in the tunica intima layer of the rat IA model and that TNF‐α mediated the activation of EGFR in VSMCs in vitro. These results indicated that ECs may affect the phenotypic modulation of VSMCs by regulating the expression of TNF‐α. In addition, other studies have shown that ECs can secrete EGFR ligands, thus affecting angiogenesis,^45^, ^46^ but whether ECs can affect the phenotype of VSMCs by secreting EGFR ligands remains unelucidated. Furthermore, whether erlotinib can regulate the function of ECs remains to be determined.

Epidermal growth factor receptor activation is generally mediated by specific ligands such as EGF, HB‐EGF, and EREG. As expected, our results confirmed the upregulation of EGFR ligands in aneurysm models. However, many other factors—including abnormal hemodynamics, pro‐inflammatory cytokines, and metabolites—may also induce VSMC phenotypic modulation through EGFR activation. Our data additionally signified that TNF‐α expression increased in rat IA models; and in vitro study indicated that EGFR was activated in TNF‐α‐stimulated, cultured VSMCs. These results suggested that in cultured VSMCs, EGFR not only transduced phenotypic modulation signals from its cognate ligands but also from other non‐EGFR ligands. EGFR activation by non‐EGFR ligands is termed receptor transactivation, which has recently been reported to occur in rat aortic SMCs.^47^ As a pro‐inflammatory factor, TNF‐α has been reported to play an important role in the development of IA, although a regulatory effect on EGFR has not yet been described^20^; thus, our study can act as a supplement to the previously cited study.

Inflammation is an important mechanism in the initiation and maintenance of IA, and previous studies revealed that in addition to ECs and VSMCs, inflammatory cells that include macrophages and lymphocytes are also associated with the development of IA.^48^ Modulation of the VSMC phenotype into an inflammatory state was uncovered during the initiation of IA prior to inflammatory cell infiltration.^48^ In accordance with this, we observed increased expression of MMPs and TNF‐α in the rat IA models and VSMCs in the WSS‐stimulated co‐culture model. EGFR inhibition attenuated the expression of MMPs, suggesting that EGFR regulates the production of cytokines in VSMCs. In addition, there exists the possibility that EGFR affects the production of cytokines in other cells such as macrophages, given that macrophages have been observed in IA injury.^49^

In summary, our results suggest that abnormal hemodynamics activate EGFR in VSMCs at the early stages of IA formation and stimulate VSMC phenotypic modulation leading to the initiation of IA. However, pharmacological blockade of EGFR by erlotinib attenuated IA formation and phenotypic modulation of VSMCs. Further study revealed that EGFR was transactivated by non‐EGFR ligands such as TNF‐α. This study thus exposes the potential use of EGFR inhibitors in the treatment of IA and provides new avenues for its prevention.

## CONFLICT OF INTEREST

The authors declare no conflict of interest with respect to the research, authorship, and publication of this article.

## AUTHOR CONTRIBUTIONS

Y. L. designed the experiments, analyzed the data, and wrote the manuscript. H. T performed the in vitro experiments and part of the animal experiments. Z. Z. performed the rest of animal experiments. R. Z, C. W., and Q. H. contributed to human sample collection. W. H. and J. L. are corresponding authors.

## Supporting information

Supplement S1

Supplement S2

Supplement S3

## Data Availability

The datasets generated during and/or analyzed during the current study are available from the corresponding author on reasonable request.
