# Supplementary material for: Pharmacological inhibition of epidermal growth factor receptor attenuates intracranial aneurysm formation by modulating the phenotype of vascular smooth muscle cells
Source: CNS Neurosci Ther. 2021 Nov 2;28(1):64–76. doi: 10.1111/cns.13735 (PMC8673708; doi:10.1111/cns.13735)
Supplement: Supplementary file 1 — Supplement S1 [file CNS-28-64-s002.pdf]

Supporting information

**Table S1.** Incidence of IA in each group according to statistical analysis

| Groups | Grade 0 | Grade 1 | Grade 2 | Grade 3 | Kruskal-Wallis test |
|---|---|---|---|---|---|
| Sham | 10 | 0 | 0 | 0 | $P \leq 0.001$(H = 22.26) |
| IA | 0 | 1 | 6 | 3 | |
| IA + erlotinib | 3 | 5 | 2 | 0 | |

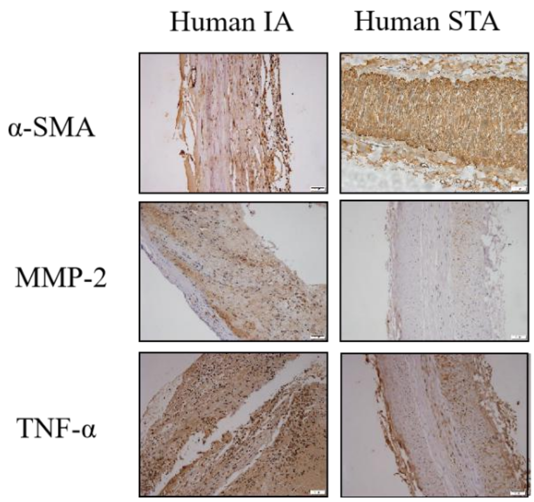


**Figure S1.** Tissue samples were from unruptured human IA (left) and human STA (right). The tissue sections were stained for α-SMA (brown), MMP-2 (brown) and TNF-α (brown) by IHC. Representative images were shown.

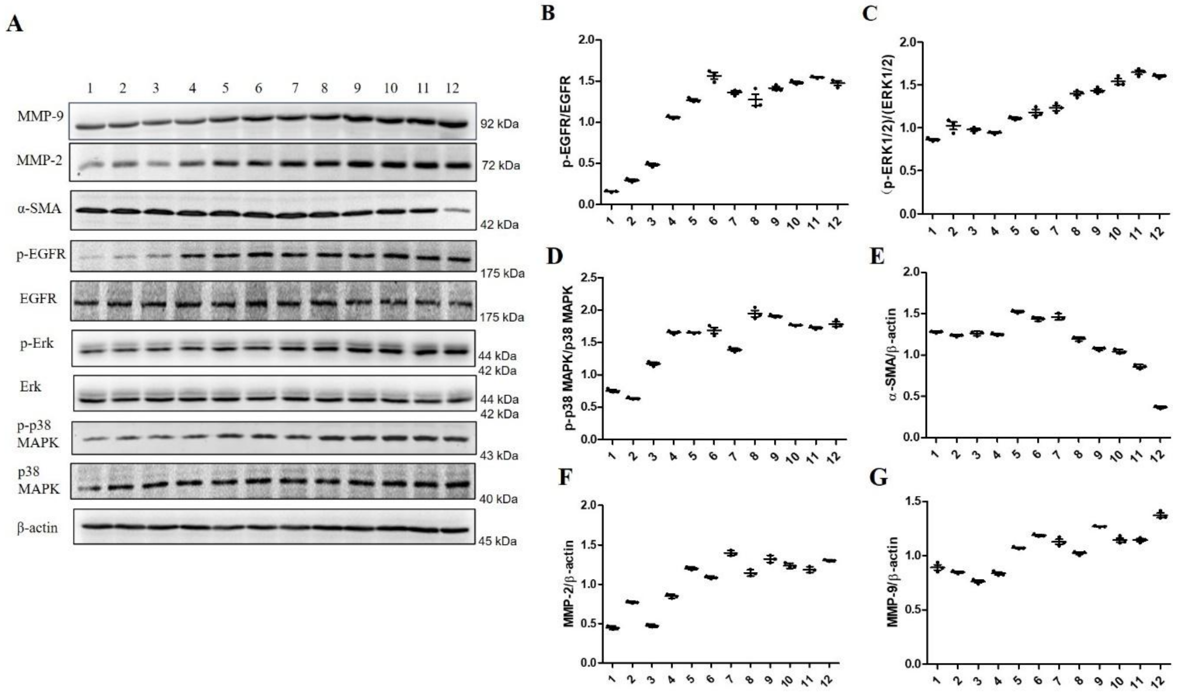


**Figure S2.** EGFR activation and downstream signals of VSMCs in *in vitro* co-culture cell models with stimulation of hemodynamics. **A:** Expression levels of p-EGFR, EGFR, p-Erk1/2, Erk1/2, p-p38MAPK, p38MAPK, α-SMA, MMP-2 and MMP-9 with the extension of stimulation time and strength of WSS. (1: 0 dyn/cm$^2$ + 24h; 2: 0 dyn/cm$^2$ + 48h; 3: 0 dyn/cm$^2$ + 72h; 4: 4 dyn/cm$^2$ + 24h; 5: 4 dyn/cm$^2$ + 48h; 6: 4 dyn/cm$^2$ + 72h; 7: 12 dyn/cm$^2$ + 24h; 8: 12 dyn/cm$^2$ + 48h; 9: 12dyn/cm$^2$ + 72h; 10: 36 dyn/cm$^2$ + 24h; 11: 36 dyn/cm$^2$ + 48h; 12: 36 dyn/cm$^2$ +72h). **B, C, and D:** The expression levels of p-EGFR, p-Erk and p-p38 MAPK were quantified and normalized by their unphosphorylated forms. **E, F and G:** The expression of α-SMA, MMP-2 and MMP-9 were quantified and normalized by β-actin. Data are represented as the mean ± SD.

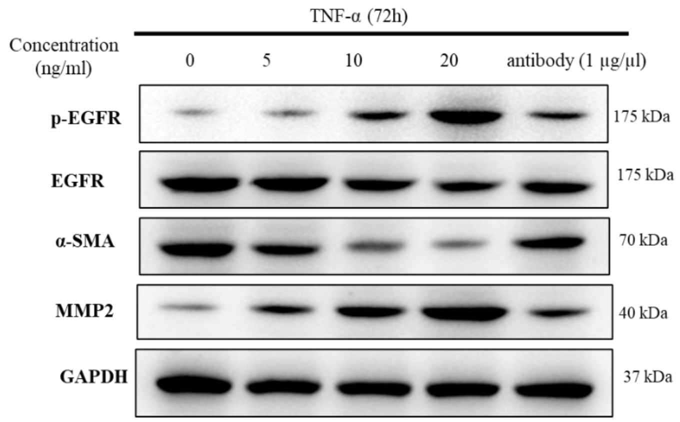


**Figure S3**. Protein expression of p-EGFR, EGFR, α-SMA and MMP-2 in VSMCs stimulated by TNF-α (20 ng/μl) with different concentrations and TNF-α antibody (1 μg/μl) analyzed by western blot.
