# Supplementary material for: Pharmacological inhibition of epidermal growth factor receptor attenuates intracranial aneurysm formation by modulating the phenotype of vascular smooth muscle cells
Source: CNS Neurosci Ther. 2021 Nov 2;28(1):64–76. doi: 10.1111/cns.13735 (PMC8673708; doi:10.1111/cns.13735)
Supplement: Supplementary file 2 — Supplement S2 [file CNS-28-64-s001.pdf]

Full unedited blots for Figure 2A

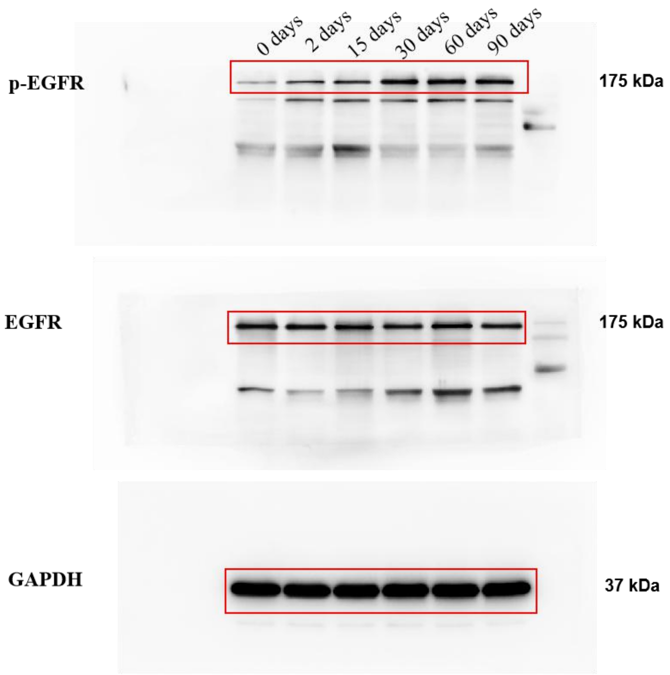

Full unedited blots for Figure 2D

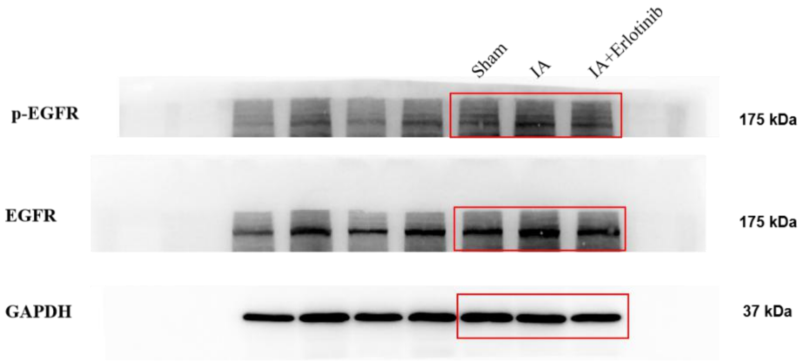

Full unedited blots for Figure 3A

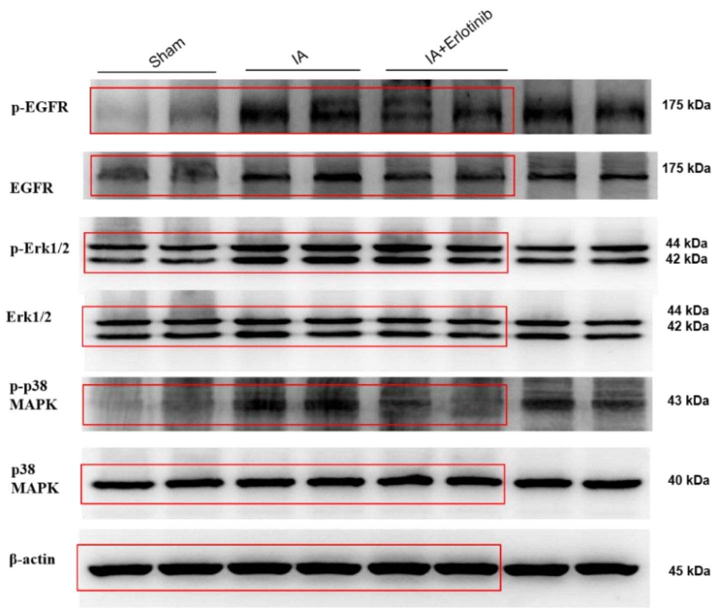

Full unedited blots for Figure 5B

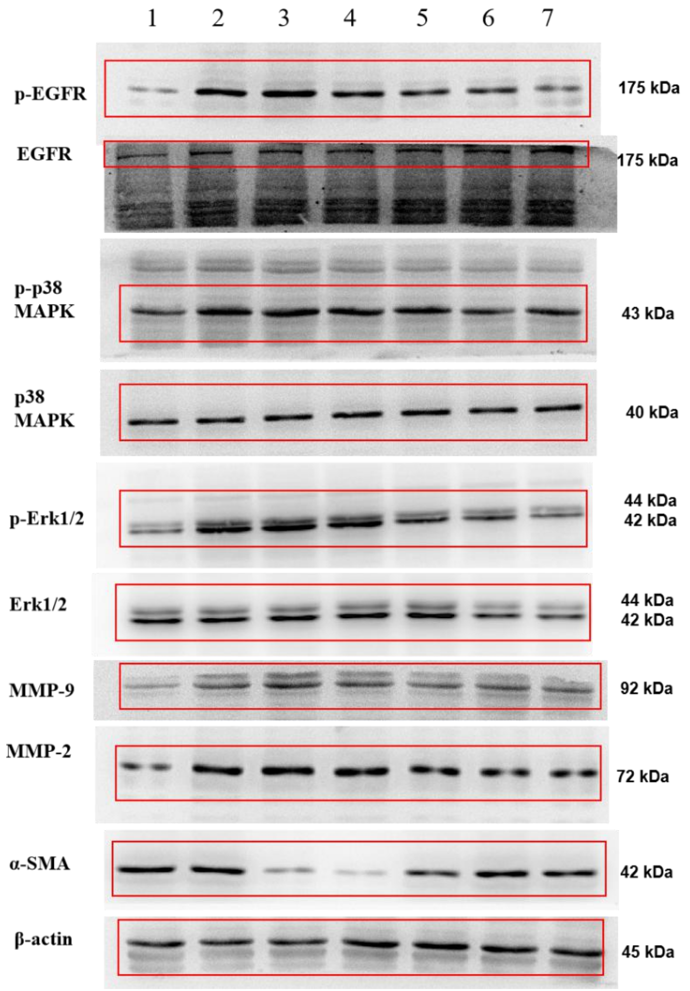

Full unedited blots for Figure 6D

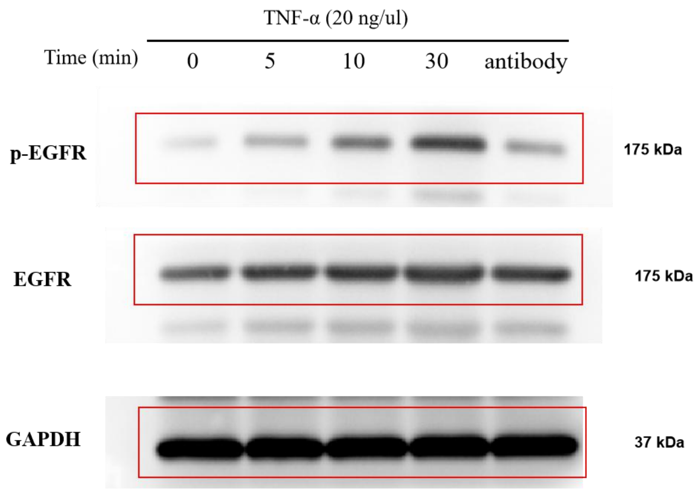

Full unedited blots for SI Figure 2A

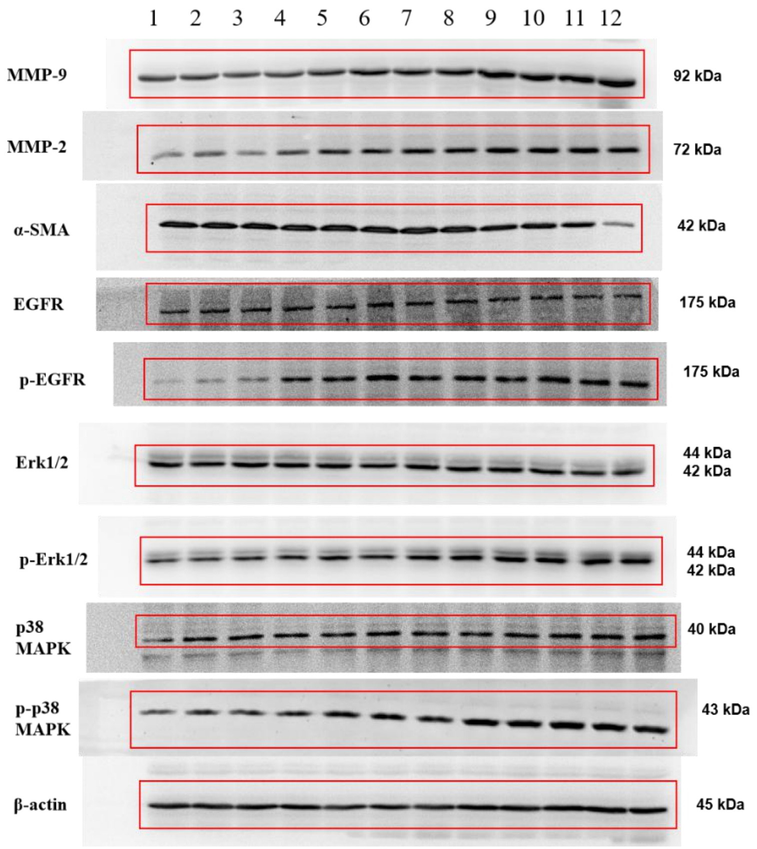

Full unedited blots for SI Figure 3

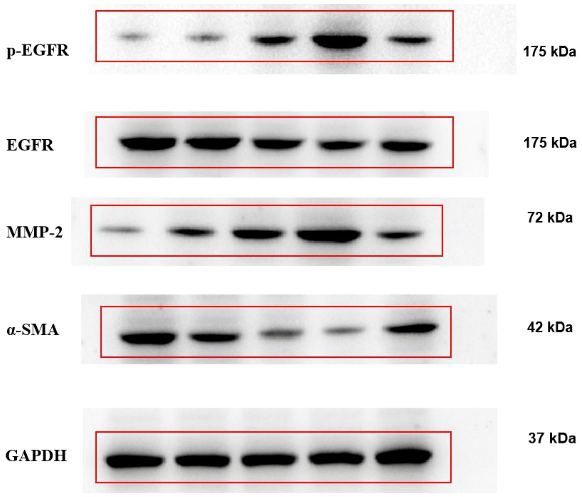
p-EGFR
175 kDa
EGFR
175 kDa
MMP-2
72 kDa
α-SMA
42 kDa
GAPDH
37 kDa
