# Supplementary figures and images for: Pharmacological inhibition of epidermal growth factor receptor attenuates intracranial aneurysm formation by modulating the phenotype of vascular smooth muscle cells
Source: CNS Neurosci Ther. 2021 Nov 2;28(1):64–76. doi: 10.1111/cns.13735 (PMC8673708; doi:10.1111/cns.13735)

High magnification pics of Figure 4C

C

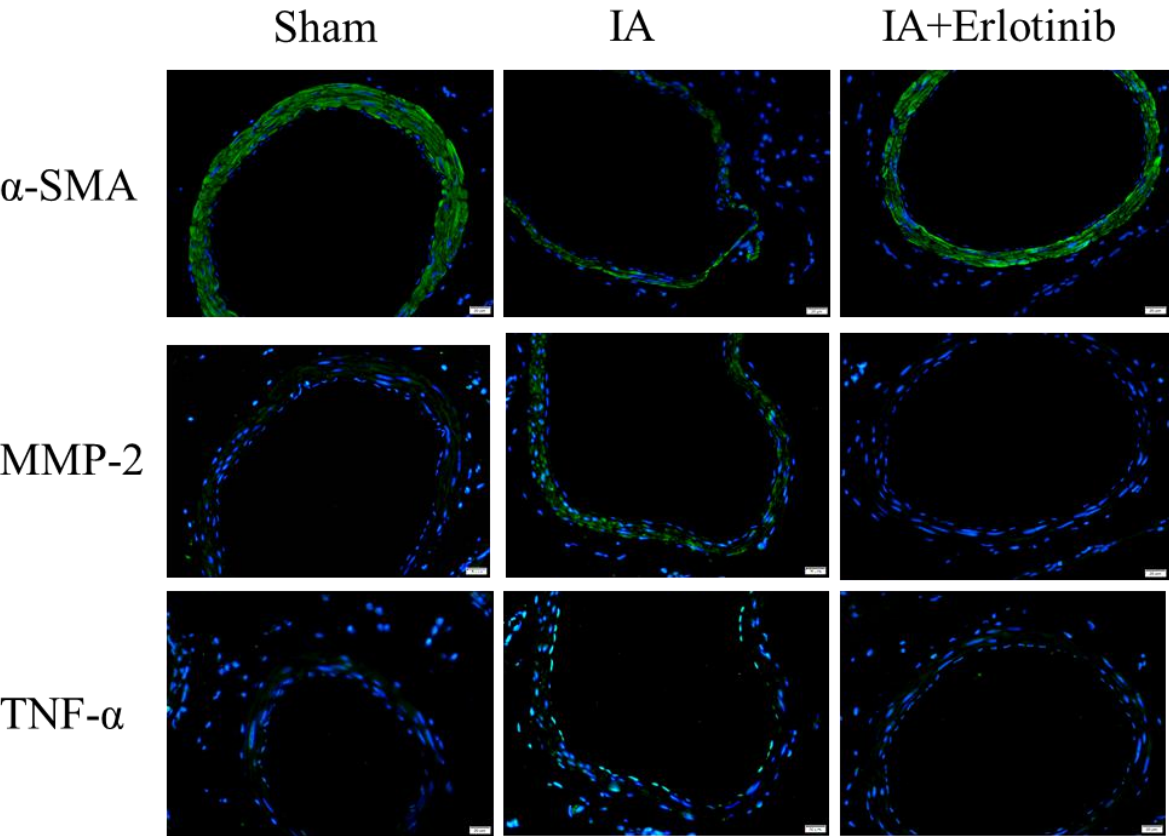

Supplement: Supplementary file 3 — Supplement S3 [file CNS-28-64-s003.pdf]
